# The Place of Nephrectomy in the Treatment of Emphysematous Pyelonephritis in the Light of Current Literature

**DOI:** 10.5152/tud.2025.24185

**Published:** 2025-12-05

**Authors:** Akif Erbin, Cagri Sevik, Halil Lutfi Canat

**Affiliations:** Department of Urology, Çam Sakura City Hospital, Istanbul, Türkiye

To the Editor,

With great interest, we have read the study titled “Scoring System to Personalize Management of Emphysematous Pyelonephritis.”^1^ Based on seven risk factors, including kidney stones, leukocytes, high creatinine, systemic inflammatory response syndrome, septic shock, urine culture, and emphysematous pyelonephritis (EPN) degree, the authors developed a novel scoring system. The authors suggested that they could treat patients classified as good-risk with conservative antibiotics, while those in the intermediate-risk category could undergo treatment with DJ stent placement, either with or without percutaneous drain (PCD). Patients in the poor-risk group may require interventions such as percutaneous nephrostomy placement ± PCD placement. Nonetheless, no patient received an initial nephrectomy without prior intervention.

Currently, the treatments for EPN have shifted from invasive procedures to more conservative methods, presumably attributable to breakthroughs in imaging techniques, antibiotic therapy, drainage technologies, and new scoring systems.^2^ In 2000, Huang and Tseng^3^ introduced a classification based on computed tomography findings, which is extensively utilized in clinical evaluations. In Class 1, gas is confined to the renal collecting system; in Class 2, gas is located within the renal parenchyma, without extending to the extrarenal region; in Class 3A, gas or abscesses are found in the perinephric space; in Class 3B, gas or abscesses are present in the pararenal space; and in Class 4, bilateral involvement is evident.^3^ An escalation in gas distribution reflects a corresponding expansion of the infection zone, which correlates with an increase in the mortality rate. A recent multicentric (15 centers, 570 patients) study proposed an 8-point scoring system that incorporates the parameter of paranephric gas extension (Class 3B). The scoring criteria consisted of a quick sepsis-related organ failure diagnosis score ≥2 (2 points), anemia, paranephric gas extension, leukocyte count >22 000/μL, thrombocytopenia, and hyperglycemia (1 point each). The mortality rate was less than 5% for scores of 3 or lower, 83.3% for a score of 6, and 100% for a score of 7, and the area under the curve for this scoring system was 0.90.^4^

In conclusion, in patients classified as Class 3B or higher, the presence of risk factors necessitates urgent nephrectomy rather than conservative interventions. In the study presented, a conservative approach was also applied to the poor prognostic group. The study featured a brief follow-up period; particularly in instances of recurrence during mid-term or long-term follow-up, the risk of mortality may be increased.

## Data Availability

The data that support the findings of this study are available on request from the corresponding author.
